# Evaluating private hospital performance from intellectual capital and digital perspective

**DOI:** 10.1097/MD.0000000000041086

**Published:** 2024-12-20

**Authors:** Xiaoming Chen, Xin Shan, Jian Xu

**Affiliations:** a School of Economics and Management, Qingdao Agricultural University, Qingdao, China.

**Keywords:** digital transformation, human capital, intellectual capital, private hospital performance, relational capital, structural capital

## Abstract

The objective of this study is to examine the impact of intellectual capital (IC) and its elements on private hospital performance. In addition, this study explores the moderating role of digital transformation in the relationship between IC and its elements and private hospital performance. This study selects 13 private listed hospitals from 2012 to 2022 as the sample. IC is assessed by the modified value added intellectual coefficient model, hospital performance is measured by financial performance and sustainable growth, and digital transformation is measured using text analysis. Multiple regression analysis is used to do the analyses. The results show that IC and only one element (structural capital) have a positive impact on private hospital performance, while human and relational capitals have no significant impact. Surprisingly, digital transformation negatively moderates the relationship between IC and private hospital performance. This study underscores the pivotal role of IC in enhancing the performance of private hospitals, and hospital managers should accelerate hospital digital transformation in the digital era.

## 1. Introduction

Intellectual capital (IC) is the valuable intangible asset in hospitals,^[1,2]^ consisting of human capital (HC), structural capital (SC), and relational capital (RC).^[3–7]^ HC refers to the knowledge, skills, and experience of hospital employees.^[8]^ Through continuous professional training, academic exchanges, and practical experience accumulation, doctors, nurses, and other medical staff can enhance their professional skills and provide more accurate and efficient medical services to patients. SC involves hospital organizational structure, processes, patents, information systems, and the like.^[9]^ Hospitals can achieve a rational resource allocation and improve operational efficiency by optimizing organizational structure, improving management systems, and introducing advanced information systems. In addition, SC can reduce the operational costs of hospitals. RC encompasses the trust and interaction between the hospital, its patients, partners, and the community.^[10]^ By establishing strong relationships with patients, partners, and the community, hospitals can build a positive brand image, which can not only attract more patients, but also bring more business opportunities. Meanwhile, hospitals can enhance their market competitiveness by collaborating with other medical institutions. Therefore, IC is an important factor of improving hospital performance.

The latest disruptive technologies have changed the healthcare environment, and the quality of care has been rapidly improved.^[11]^ The successful digital transformation in hospitals can save lives and provide doctors with a convenient working environment through the application of digital technologies.^[12]^ For example, telemedicine empowers patients in remote and distant locations to get the same life-saving support as their counterparts, and it saves doctors a lot of time. Artificial intelligence can help doctors achieve faster medicine trials. Therefore, digital transformation plays an important role in improving hospital services, thus enhancing profitability.

Private hospitals play a significant role in China’s medical system. As an important supplement to public hospitals, private hospitals alleviates the difficulty in accessing medical services in areas with relatively scarce medical resources.^[13]^ Private hospitals tend to focus more on innovation and introduce new technologies and treatments quickly, which brings new opportunities for the entire medical industry. They attach great importance to medical quality and patient experience and win the trust of patients by providing personalized medical services. In addition, they can create more job opportunities for society and promote local economic development. However, private hospitals face some challenges, such as talent shortages, insufficient funds, and policy restrictions.^[14]^ Therefore, private hospitals need to improve the value creation efficiency of IC assets to gain competitive advantage in the fierce market competition.

The current literature has examined the impact of IC on organization performance with various results.^[3,15,16]^ For example, Nguyen^[17]^ found that IC and HC contribute to firm performance in Vietnam. Ayinaddis et al^[18]^ observed a positive relationship between IC and HC and financial performance of insurance companies and a nonsignificant relationship between SC and RC and financial performance. The study by Qomariah et al^[19]^ showed that IC has no impact on firm profitability in pharmaceutical companies during COVID-19. In addition, very few studies have focus on how IC improves hospital performance. Harris^[20]^ found that nursing IC is associated with hospital performance. Jang et al^[21]^ found that the increased IC contributes to hospital performance in Korea. Chung^[22]^ argued that the performance of nurses in medium and small hospitals is closely related to the investment in IC. Therefore, analyzing the role of IC is essential in helping hospital managers enhance their performance.

The aim of this study is to investigate the role of IC and its components in improving private hospital performance. In addition, we examine the moderating role of digital transformation. This study selects private listed hospitals in the Chinese A-share market as the sample, and use the modified value added intellectual coefficient (MVAIC) model to measure IC. Hospital performance is measured by financial returns and sustainable development capability, and hospital digital transformation level is measured by text analysis.

The contributions of this study are presented in 3 aspects. First, previous research on IC has paid less attention to the health sector, and this is the first study that examine the impact of IC on private hospital performance, which could expand the existing literature. Second, this study unveils the moderating impact of digital transformation between IC and private hospital performance. Finally, this study can help hospital managers to strengthen the management of IC resources and accelerate digital transformation of hospitals.

## 2. Methods

### 2.1. Sample

The sample consists of private listed hospitals in the Chinese A-share market. We collect relevant data from 2012 to 2022. Digital transformation was first proposed in 2012, and the year 2012 was chosen as the starting point. After screening, we delete hospitals with missing data, delisted hospitals, and special treatment hospitals. Finally, we get 114 observations for 13 private hospitals. The data are retrieved from the CSMAR database, and Stata 17.0 is used to analyze the data. In this study, the approval of IRB is not required.

### 2.2. Variable definition

**Figure 1.**
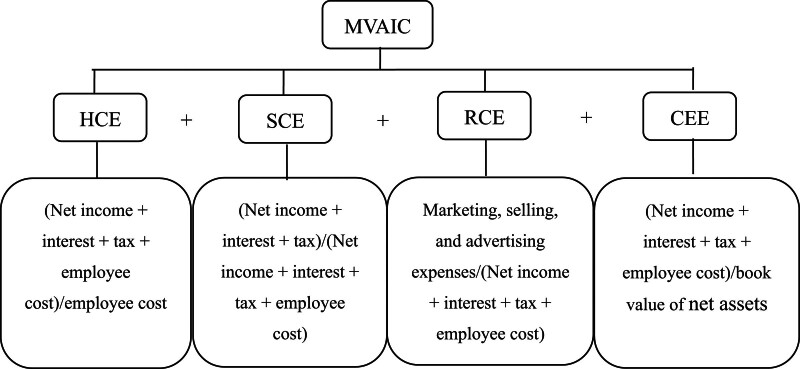
Calculation process. CEE = capital employed efficiency, HCE = human capital efficiency, MVAIC = modified value added intellectual coefficient, RCE = relational capital efficiency, SCE = structural capital efficiency.

1. *Dependent variables*. Referring to Xu and Wang,^[15]^ in this study, hospital performance is evaluated from 2 aspects: financial performance and sustainable growth. Financial performance is measured by return on assets (ROA) and return on equity (ROE), consistent with previous studies.^[3,23,24]^ Sustainable growth is measured by sustainable growth rate (SGR) based on Higgins sustainable growth model.^[15,24,25]^
2. *Independent variables*. Guided by Xu and Li,^[3]^ Xu and Zhang,^[26]^ and Gao et al,^[27]^ the MVAIC model is used to measure IC. This model computes MVAIC based on 4 elements: HC efficiency (HCE), SC efficiency (SCE), RC efficiency, and capital employed efficiency (CEE). The calculation process is listed in Figure 1.
3. *Moderating variable*. Referring to previous literature,^[28–30]^ we use text analysis to measure the level of hospital digital transformation (DIG).
4. *Control variables*. Consistent with previous literature,^[3–7,15–18,26]^ hospital size (SIZE), leverage ratio (LEV), sales growth rate (SALES), and hospital age (AGE) are chosen. In addition, we include firm fixed effects and year fixed effects in all models. Table 1 shows the definition of variables.

**Table 1:** Variable definition.

Variable	Symbol	Measurement
Return on assets	ROA	Net income/total assets
Return on equity	ROE	Net income/total shareholders’ equity
Sustainable growth rate	SGR	Net profit ratio × asset turnover ratio × retention ratio × equity multiplier
Modified value added intellectual coefficient	MVAIC	CEE + HCE + SCE + RCE
Capital employed efficiency	CEE	(Net income + interests + taxes + employee costs)/book value of net assets
Human capital efficiency	HCE	(Net income + interests + taxes + employee costs)/employee costs
Structural capital efficiency	SCE	1-employee costs/(net income + interests + taxes + employee costs)
Relational capital efficiency	RCE	Marketing, selling and advertising expenses/(Net income + interests + taxes + employee costs)
Digital transformation	DIG	Natural logarithm of the number of keywords plus 1
Hospital size	SIZE	Natural logarithm of total assets
Leverage ratio	LEV	Total liabilities/total assets
Sales growth rate	SALES	Sales in current period/Sales in previous period-1
Hospital age	AGE	Natural logarithm of hospital age since the setup of hospital

### 2.3. Models

Table 2 shows all models used in this study. Model (1) examines the impact of IC on private hospital performance, and Model (2) examines the relationship between IC elements and private hospital performance. Models (3) and (4) investigates the moderating effect of digital transformation between IC and its elements and hospital performance. Firm performance includes ROA, ROE, and SGR.

**Table 2:** Model specification.

No.	Model
1	PERF = β_0_ + β_1_MVAIC + β_2_SIZE + β_3_LEV + β_4_SALES + β_5_AGE + ∑FIRM + ∑YEAR + ε
2	PERF = β_0_ + β_1_CEE + β_2_HCE + β_3_SCE + β_4_RCE + β_5_SIZE + β_6_LEV + β_7_SALES + β_8_AGE + ∑FIRM + ∑YEAR + ε
3	PERF = β_0_ + β_1_MVAIC + β_2_DIG + β_3_MVAIC × DIG + β_4_SIZE + β_5_LEV + β_6_SALES + β_7_AGE + ∑FIRM + ∑YEAR + ε
4	PERF = β_0_ + β_1_CEE + β_2_HCE + β_3_SCE + β_4_RCE + β_5_DIG + β_6_CEE × DIG + β_7_HCE × DIG + β_8_SCE × DIG + β_9_RCE × DIG + β_10_SIZE + β_11_LEV + β_12_SALES + β_13_AGE + ∑FIRM + ∑YEAR + ε

AGE = hospital age, CEE = capital employed efficiency, DIG = digital transformation, FIRM = firm fixed effects, HCE = human capital efficiency, LEV = leverage ratio, MVAIC = modified value added intellectual coefficient, PERF = firm performance, RCE = relational capital efficiency, SALES = sales growth rate, SCE = structural capital efficiency, SIZE = hospital size, YEAR = year fixed effects.

## 3. Results

### 3.1. Descriptive statistics

Table 3 presents the descriptive statistics. The mean values of ROA and ROE are 0.013 and ‐0.285, respectively, suggesting that private hospitals have lower profitability. This could be caused by the outbreak of COVID-19. It is estimated that 8 out of 13 private hospitals experienced losses during the COVID-19, supporting the findings of Wang and Xu.^[13]^ SGR has a mean value of 0.008, and the lower profitability leads to lower SGR. MVAIC has a mean value of 2.526, indicating that private hospitals invest 1 yuan in IC, and it will create 2.526 yuan. Among IC elements, HC has the highest mean value, followed by SC and RC. The DIG mean is 1.405, and its standard deviation is 1.337, suggesting a great difference in hospital digital transformation level. The mean value of LEV (0.420) means a good capital structure of private hospitals.

**Table 3:** Descriptive statistics.

Variable	Mean	Medium	Maximum	Minimum	SD
ROA	0.013	0.029	0.307	‐0.509	0.122
ROE	‐0.285	0.052	0.388	‐20.992	2.267
SGR	0.008	0.044	0.559	‐0.955	0.211
MVAIC	2.526	1.980	41.642	‐12.478	5.675
CEE	0.349	0.221	15.108	‐9.368	1.801
HCE	1.298	0.589	27.394	‐2.537	3.927
SCE	0.477	0.634	8.098	‐7.386	1.688
RCE	0.401	0.200	15.196	‐1.309	1.509
DIG	1.405	1.099	4.466	0	1.337
SIZE	21.842	21.700	24.003	20.044	0.901
LEV	0.420	0.379	0.990	0.033	0.225
SALES	0.193	0.096	4.566	‐0.632	0.576
AGE	2.864	2.890	3.434	2.197	0.274

AGE = hospital age, CEE = capital employed efficiency, DIG = digital transformation, HCE = human capital efficiency, LEV = leverage ratio, MVAIC = modified value added intellectual coefficient, RCE = relational capital efficiency, ROA = return on assets, ROE = return on equity, SALES = sales growth rate, SCE = structural capital efficiency, SGR = sustainable growth rate, SIZE = hospital size.

### 3.2. Correlation analysis

Table 4 shows the results of correlation analysis. MVAIC is found to be related to ROE, and DIG is not correlated with ROA, ROE, and SGR. CEE and SCE are positively related to ROA, ROE, and SGR, while HCE is negatively related to ROA and SGR. In addition, we find that all values of variance inflation factor are <5, indicating no serious multicollinearity problem.

**Table 4:** Correlation matrix.

Variable	1	2	3	4	5	6	7	8	9	10	11	12	13
1 ROA	1												
2 ROE	0.469***	1											
3 SGR	0.900***	0.650***	1										
4 MVAIC	0.148	0.283***	0.079	1									
5 CEE	0.263***	0.519***	0.208**	0.360***	1								
6 HCE	‐0.163*	0.059	‐0.196**	0.872***	0.008	1							
7 SCE	0.721***	0.235**	0.649***	0.222**	0.131	‐0.140	1						
8 RCE	‐0.136	0.028	‐0.167*	0.812***	-0.006	0.825***	‐0.077	1					
9 DIG	‐0.069	‐0.049	‐0.079	0.070	0.017	0.126	‐0.158*	0.092	1				
10 SIZE	0.035	‐0.040	‐0.003	0.033	0.021	0.052	‐0.015	‐0.017	0.495***	1			
11 LEV	‐0.363***	‐0.351***	‐0.462***	0.109	0.116	0.139	‐0.226**	0.160*	0.081	0.281***	1		
12 SALES	0.065	0.055	0.079	0.171*	‐0.064	0.162*	0.043	0.251***	0.049	0.106	‐0.121	1	
13 AGE	‐0.093	‐0.055	‐0.063	0.086	0.054	0.157*	‐0.227**	0.103	0.486***	0.382***	0.116	0.016	1

AGE = hospital age, CEE = capital employed efficiency, DIG = digital transformation, HCE = human capital efficiency, LEV = leverage ratio, MVAIC = modified value added intellectual coefficient, RCE = relational capital efficiency, ROA = return on assets, ROE = return on equity, SALES = sales growth rate, SCE = structural capital efficiency, SGR = sustainable growth rate, SIZE = hospital size.

* *P* < .10.

** *P* < .05.

*** *P* < .01.

### 3.3. Regression results

Table 5 shows the regression results of Model (1). The coefficients of MVAIC are positive and significant, which implies that IC can promote hospital performance improvement. Regarding control variables, LEV has a negative impact on private hospital performance, while other variables have no significant impact.

**Table 5:** Regression results of Model (1).

Variable	ROA	ROE	SGR
Constant	0.235 (0.28)	‐8.059 (‐0.38)	0.569 (0.36)
MVAIC	0.005** (3.51)	0.158** (4.19)	0.007* (2.62)
SIZE	0.019 (1.02)	0.739 (1.56)	0.053 (1.50)
LEV	‐0.285** (‐4.74)	‐3.308* (‐2.19)	‐0.564** (‐5.00)
SALES	‐0.020 (‐1.32)	‐0.442 (‐1.13)	‐0.029 (‐1.00)
AGE	‐0.169 (‐0.73)	‐2.433 (‐0.42)	‐0.499 (‐1.16)
FIRM	Yes	Yes	Yes
YEAR	Yes	Yes	Yes
Adj. *R*^2^	0.5421	0.1646	0.4630
*F*	5.95**	1.82*	4.61**

AGE = hospital age, FIRM = firm fixed effects, LEV = leverage ratio, MVAIC = modified value added intellectual coefficient, ROA = return on assets, ROE = return on equity, SALES = sales growth rate, SGR = sustainable growth rate, SIZE = hospital size, YEAR = year fixed effects.

* *P* < .05. *t*-values are in parentheses.

** *P* < .01. *t*-values are in parentheses.

Table 6 presents the regression results of Model (2). The coefficients of CEE are positive and significant, suggesting that physical capital plays an important role in improving private hospital performance. SC has a positive impact on ROA and SGR. Contrary to most literature,^[3,4,10,15,17,18,22–24,26]^ HC is found to have no impact on hospital performance. In addition, RC has no impact on private hospital performance.

**Table 6:** Regression results of Model (2).

Variable	ROA	ROE	SGR
Constant	‐0.542 (-0.96)	‐3.727 (‐0.21)	‐0.574 (‐0.44)
CEE	0.012*** (3.86)	0.747*** (7.53)	0.022*** (3.06)
HCE	0.002 (0.69)	0.120 (1.45)	‐0.002 (‐0.40)
SCE	0.042*** (10.22)	‐0.029 (‐0.22)	0.057*** (6.06)
RCE	‐0.005 (‐0.68)	0.007 (0.03)	0.005 (0.32)
SIZE	0.019 (1.50)	0.848** (2.15)	0.054* (1.89)
LEV	‐0.117** (‐2.58)	‐4.284*** (‐2.98)	‐0.353*** (‐3.37)
SALES	‐0.001 (‐0.06)	‐0.162 (‐0.48)	‐0.003 (‐0.14)
AGE	0.069 (0.44)	‐4.619 (‐0.94)	‐0.160 (‐0.45)
FIRM	Yes	Yes	Yes
YEAR	Yes	Yes	Yes
Adj. *R*^2^	0.8015	0.4206	0.6452
*F*	16.21***	3.73***	7.85***

AGE = hospital age, CEE = capital employed efficiency, FIRM = firm fixed effects, HCE = human capital efficiency, LEV = leverage ratio, RCE = relational capital efficiency, ROA = return on assets, ROE = return on equity, SALES = sales growth rate, SCE = structural capital efficiency, SGR = sustainable growth rate, SIZE = hospital size, YEAR = year fixed effects.

* *P* < .10. *t*-values are in parentheses.

** *P* < .05. *t*-values are in parentheses.

*** *P* < .01. *t*-values are in parentheses.

Table 7 shows the regression results of Model (3). When ROA and SGR are the dependent variables, the coefficients of MVAIC × DIG are negative and significant, which suggests that digital transformation negatively moderates the relationship between IC and hospital performance.

**Table 7:** Regression results of Model (3).

Variable	ROA	ROE	SGR
Constant	‐0.231 (‐0.28)	‐12.116 (‐0.56)	‐0.125 (‐0.08)
MVAIC	0.017*** (3.47)	0.297** (2.27)	0.023** (2.42)
DIG	0.022** (2.13)	0.122 (0.44)	0.038* (1.90)
MVAIC × DIG	‐0.005** (‐2.48)	‐0.061 (‐1.10)	‐0.007* (‐1.68)
SIZE	0.020 (1.09)	0.782 (1.62)	0.052 (1.47)
LEV	‐0.253*** (‐4.03)	‐2.767* (‐1.68)	‐0.534*** (‐4.45)
SALES	‐0.019 (‐1.28)	‐0.408 (‐1.03)	‐0.029 (‐1.01)
AGE	‐0.026 (‐0.11)	‐1.479 (‐0.25)	‐0.267 (‐0.61)
FIRM	Yes	Yes	Yes
YEAR	Yes	Yes	Yes
Adj. *R*^2^	0.5753	0.1573	0.4824
*F*	6.28***	1.73**	4.63***

AGE = hospital age, DIG = digital transformation, FIRM = firm fixed effects, LEV = leverage ratio, MVAIC = modified value added intellectual coefficient, ROA = return on assets, ROE = return on equity, SALES = sales growth rate, SGR = sustainable growth rate, SIZE = hospital size, YEAR = year fixed effects.

* *P* < .10. *t*-values are in parentheses.

** *P* < .05. *t*-values are in parentheses.

*** *P* < .01. *t*-values are in parentheses.

In Table 8, we find that DIG has no impact on private hospital performance. The coefficients of the interaction CEE × DIG are significant and negative, suggesting a negative moderating effect of digital transformation in the relationship between physical capital and private hospital performance. When ROA is the dependent variable, the coefficient of SCE × DIG is significant and negative, which suggests a negative moderating effect of digital transformation in the relationship between SC and financial performance. The coefficients of HCE × DIG and RC efficiency × DIG are not significant, which means that digital transformation has no moderating effect.

**Table 8:** Regression results of Model (4).

Variable	ROA	ROE	SGR
Constant	‐1.028* (‐1.87)	‐7.784 (‐0.40)	‐1.445 (‐1.06)
CEE	0.051*** (4.96)	1.470*** (4.03)	0.093*** (3.60)
HCE	0.006 (0.87)	0.082 (0.32)	‐0.003 (‐0.17)
SCE	0.051*** (8.58)	‐0.018 (‐0.08)	0.068*** (4.60)
RCE	‐0.011 (‐0.37)	0.675 (0.64)	0.003 (0.04)
DIG	0.010 (1.44)	0.140 (0.58)	0.024 (1.39)
CEE × DIG	‐0.017*** (‐3.93)	‐0.306** (‐2.06)	‐0.030*** (‐2.84)
HCE × DIG	‐0.002 (‐0.74)	0.006 (0.06)	‐0.0002 (‐0.03)
SCE × DIG	‐0.005** (‐2.30)	‐0.016 (‐0.22)	‐0.006 (‐1.20)
RCE × DIG	0.003 (0.26)	‐0.257 (‐0.64)	0.002 (0.08)
SIZE	0.027** (2.33)	0.928** (2.26)	0.067** (2.30)
LEV	‐0.109** (‐2.40)	‐4.344*** (‐2.72)	‐0.377*** (‐3.35)
SALES	‐0.001 (‐0.07)	‐0.136 (‐0.39)	‐0.007 (‐0.29)
AGE	0.169 (1.11)	‐3.884 (‐0.73)	0.050 (0.13)
FIRM	Yes	Yes	Yes
YEAR	Yes	Yes	Yes
Adj. *R*^2^	0.8393	0.4258	0.6703
*F*	17.86***	3.39***	7.56***

AGE = hospital age, CEE = capital employed efficiency, DIG = digital transformation, FIRM = firm fixed effects, HCE = human capital efficiency, LEV = leverage ratio, RCE = relational capital efficiency, ROA = return on assets, ROE = return on equity, SALES = sales growth rate, SCE = structural capital efficiency, SGR = sustainable growth rate, SIZE = hospital size, YEAR = year fixed effects.

* *P* < .10. *t*-values are in parentheses.

** *P* < .05. *t*-values are in parentheses.

*** *P* < .01. *t*-values are in parentheses.

## 4. Discussion

### 4.1. Result discussion

In this study, we find that IC has a positive impact on financial performance and sustainable growth of private hospitals. This accords with the findings of Xu and Wang,^[13]^ Lehenchuk et al,^[24]^ Xu et al,^[31]^ Rana and Hossain,^[32]^ and Balaji and Mamilla.^[33]^ Regarding IC elements, only SC positively affects financial performance and sustainable growth of private hospitals. This could be explained by the fact that effective management processes can enhance the hospital’s work efficiency and service quality and reduce unnecessary resource waste, thus improving hospital overall performance. Medical institutions should put more resources in SC construction.^[34]^ Kim and Seo^[35]^ concluded that SC has an indirect effect on financial performance of hospital in Korea. Surprisingly, we find no significant relationship between HC and hospital performance, which is not consistent with most literature.^[3,4,10,15,17,18,22–24,26]^ This could be explained by the fact that private hospitals may have the unreasonable allocation of human resources. On the other hand, it might be caused by the lack of effective training and incentive mechanisms for their employees, which hinders their enthusiasm and creativity. In addition, RC has no impact on hospital performance. This could be because private hospitals may fail to actively establish and maintain relationship networks with their stakeholders, such as patients, suppliers, partners, and government departments. The lack of such networks may prevent private hospitals from accessing important resources and information, thus negatively affecting hospital performance. Patients often seek medical treatment in public hospitals located in economically developed regions, which causes the imbalanced hospital–patient relationship. Jang et al^[36]^ pointed out that social capital is an important factor that influences the creation of IC.

Regarding the role of digital transformation, it negatively moderates the relationship between IC and financial performance and sustainable growth. This could be because digital transformation not only involves direct capital investment but also might lead to an increase in operational costs, such as system maintenance, data security assurance, employee training, and the cost of adapting to new systems, thereby affecting hospitals’ financial performance. Digital transformation requires profound changes in hospitals’ management models, business processes, and organizational structures. These changes might influence the vested interests within the hospital, trigger resistance from employees, and increase the difficulty in management. Poor management can lead to the failure of digital transformation, which in turn has a negative impact on IC and financial performance. If a hospital’s IC does not align with its digital transformation strategy, it might result in suboptimal transformation outcomes, further affecting financial performance and sustainable growth. In addition, we also find that digital transformation level has a negative moderating role in the relationship between physical capital and hospital performance. It might be explained be the fact that digital transformation requires private hospitals to invest sufficient funds in information system construction and hardware upgrades. These investments will temporarily increase the hospital’s operating costs and reduce the funds available for other physical capital investments, thereby affecting short-term financial performance. During the digital transformation process, hospitals need to redirect resources to digital construction. This adjustment in resource allocation will directly impact the hospital’s short-term financial performance and sustainable growth.

### 4.2. Practical implication

Based on the findings, we put forward the following practical implications. First, private hospitals should reasonably allocate and utilize human resources, establish a comprehensive training system and incentive mechanism, and improve the professional skills and knowledge of medical staff. Second, private hospitals should attach importance to the establishment and maintenance of RC and incorporate it into their overall development strategy. Third, they should clarify the overall objectives of digital transformation and pay attention to the combination of SC and digital transformation. In addition, private hospitals should upgrade their existing information systems, establish a hospital data center by leveraging cloud computing technology, develop intelligent triage systems by employing artificial intelligence and big data analytics, and provide patients with personalized health management plans. Finally, the government should provide financial supports and tax incentives to private hospitals, optimize the financing environment, and encourage cooperation between universities, research institutions, and private hospitals. Additionally, the government should encourage private hospitals to establish a cooperation mechanism with public hospitals.

### 4.3. Research limitation and future direction

This study may suffer from some limitations. First, our sample only includes private hospitals, and in-depth research could be carried out to compare with public hospitals. Second, we just use financial indicators to measure hospital performance, and future studies could include nonfinancial indicators to systematically measure hospital performance. In addition, in this study, IC includes 3 components, and future studies could take more components (e.g., process capital and innovation capital) into consideration.

## 5. Conclusions

This study aims to examine the impact of IC and its elements on private hospital performance. In addition, we also explore the moderating effect of digital transformation level in this relationship. IC is measured by the MVAIC model, and hospital performance is measured by financial metrics and sustainable growth. The main conclusions are summarized as follows. First, IC and SC have a positive influence on hospital performance, while HC and RC have no impact. Second, the level of hospital digital transformation has a negative moderating effect on the relationship between IC and hospital performance. This study can help hospital managers better improve hospital performance through digital transformation and IC utilization.

## Author contributions

**Conceptualization:** Xiaoming Chen, Jian Xu.

**Data curation:** Jian Xu.

**Formal analysis:** Jian Xu.

**Funding acquisition:** Jian Xu.

**Investigation:** Xiaoming Chen.

**Methodology:** Jian Xu.

**Project administration:** Jian Xu.

**Writing – original draft:** Xiaoming Chen, Xin Shan, Jian Xu.

**Writing – review & editing:** Jian Xu.
